# Chromosomal organization of multigene families and meiotic analysis in species of Loricariidae (Siluriformes) from Brazilian Amazon, with description of a new cytotype for genus *Spatuloricaria*

**DOI:** 10.1242/bio.060029

**Published:** 2023-11-09

**Authors:** Bruno Rafael Ribeiro de Almeida, Luciano Farias Souza, Thyana Ayres Alves, Adauto Lima Cardoso, Juliana Amorim de Oliveira, Talita Fernanda Augusto Ribas, Carlos Eduardo Vasconcelos Dos Santos, Luís Adriano Santos do Nascimento, Leandro Melo Sousa, Maria Iracilda da Cunha Sampaio, Cesar Martins, Cleusa Yoshiko Nagamachi, Julio Cesar Pieczarka, Renata Coelho Rodrigues Noronha

**Affiliations:** ^1^ Laboratório de Citogenética, Centro de Estudos Avançados da Biodiversidade, Instituto de Ciências Biológicas, Universidade Federal do Pará. Belém 66075-750, Pará, Brazil; ^2^ Instituto Federal de Educação, Ciência e Tecnologia do Pará. Campus Itaituba. Itaituba, 68183-300, Pará, Brazil; ^3^ Laboratório Genômica Integrativa, Instituto de Biociências, Universidade Estadual Paulista. Botucatu, CEP 18618-970, São Paulo, Brazil; ^4^ Laboratório de Óleos da Amazônia, Universidade Federal do Pará. Belém, CEP 66075-110, Pará, Brazil; ^5^ Faculdade de Ciências Biológicas, Universidade Federal do Pará, Campus de Altamira. Altamira, CEP 68372-040, Pará, Brazil; ^6^ Instituto de Estudos Costeiros, Universidade Federal do Pará, Campus Universitário de Bragança.. Bragança, CEP 68600-000, Pará, Brazil; ^7^ Laboratório de Genética e Biologia Celular, Centro de Estudos Avançados da Biodiversidade, Instituto de Ciências Biológicas, Universidade Federal do Pará. Belém 66075-750, Pará, Brazil

**Keywords:** Repetitive DNA, Meiosis, Histone, Fish, U2 snDNA

## Abstract

In the Amazon, some species of Loricariidae are at risk of extinction due to habitat loss and overexploitation by the ornamental fish market. Cytogenetic data related to the karyotype and meiotic cycle can contribute to understanding the reproductive biology and help management and conservation programs of these fish. Additionally, chromosomal mapping of repetitive DNA in Loricariidae may aid comparative genomic studies in this family. However, cytogenetics analysis is limited in Amazonian locariids. In this study, chromosomal mapping of multigenic families was performed in *Scobinancistrus aureatus*, *Scobinancistrus pariolispos* and *Spatuloricaria* sp. Meiotic analyzes were performed in *Hypancistrus zebra* and *Hypancistrus* sp. “pão”. Results showed new karyotype for *Spatuloricaria* sp. (2n=66, NF=82, 50m-10sm-6m). Distinct patterns of chromosomal organization of histone H1, histone H3 and snDNA U2 genes were registered in the karyotypes of the studied species, proving to be an excellent cytotaxonomic tool. Hypotheses to explain the evolutionary dynamics of these sequences in studied Loricariidae were proposed. Regarding *H. zebra* and *H*. sp. “pão”, we describe the events related to synapse and transcriptional activity during the meiotic cycle, which in both species showed 26 fully synapsed bivalents, with high gene expression only during zygotene and pachytene. Both *Hypancistrus* species could be used may be models for evaluating changes in spermatogenesis of Loricariidae.

## Teaser

**Summary:** Our study shows cytogenetic characteristics of Loricariidae from the Xingu River, Brazilian Amazon. Some of these fish are endangered, so this information can help conservation programs for these animals.

## INTRODUCTION

Loricariidae comprises more than 1000 species in the Neotropics (Fricke et al., 2023). In the Brazilian Amazon, some members of this family are considered endangered. Among the main causes for this fact are the destruction of habitats (especially due to the construction of dams and mining) and overexploitation by the international market of ornamental fish (Beltrão et al., 2021). Cytogenetic data are of great importance for fish conservation studies, as they contribute to the understanding of animal reproductive biology, identification of hybrid zones and help in the elaboration of management plans (Resende et al., 2021).

Chromosome mapping of multigene families can reveal excellent markers for cytotaxonomy. They are groups of related genes that originated from a common ancestor that can spread across different regions of the genome through gene duplications, transposition events, recombination and gene conversion (Eirín-López et al., 2012). Histone genes comprise a complex multigene family, with a variable number of copies grouped in one or more chromosomal regions (Hashimoto et al., 2013; Cavalcante et al., 2018). They encode five types of proteins (histones H1, H2A, H2B, H3 and H4) that constitute nucleosomes, with an important role in chromatin structure organization and epigenetic regulation of gene expression (Nagoda et al., 2005). The U2 snDNA genes are sequences that participate in spliceosome formation by actively acting in the mRNA maturation process (Colgan et al., 1998). In some organisms, snDNA U2 is associated with other multigene families, such as rDNA 45S (Silva et al., 2015), which may present different patterns of chromosomal organization and play a relevant role in the structure and evolution of sex chromosomes (Utsunomia et al., 2014). Several studies show that there are critical points for the occurrence of double strand breaks (DSB), non-homologous recombination and chromosomal reorganization in different organisms (Barros et al., 2017). In Loricariidae, the main cytogenomic studies involving multigene families focus mainly on ribosomal DNAs (Mariotto et al., 2009; Cardoso et al., 2013; Ayres-Alves et al., 2017; Barros et al., 2017; Pety et al., 2018; Glugoski et al., 2020; Santos da Silva et al., 2021; Nirchio et al., 2023). Data on the chromosomal location of histone-DNAs and U snDNA genes in this family are restricted, respectively, to the genera *Hypostomus* (Pansonato-Alves et al., 2013), *Peckoltia* (Santos da Silva et al., 2021), *Hypancistrus* (Dos Santos et al., 2023) and *Ancistrus* (Schott et al., 2022) (Table 1).

**Table 1.**
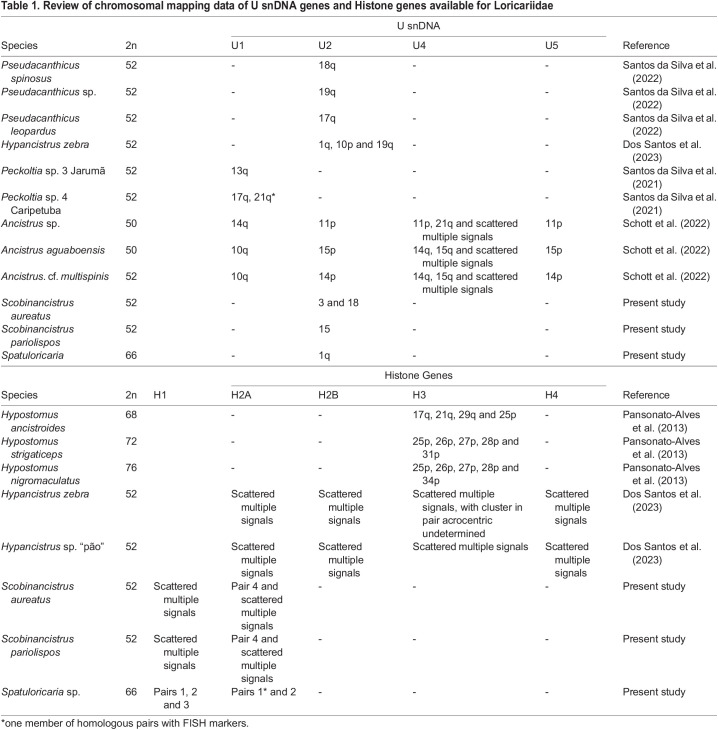
Review of chromosomal mapping data of U snDNA genes and Histone genes available for Loricariidae

Another important cytogenetic aspect to be analyzed refers to the progression of the meiotic cycle, especially the events that occur in prophase I (synapse and recombination). The study of meiosis allows identifying disorders in cell division that can affect gametogenesis (Ben Maamar et al., 2021). In most eukaryotes, the synapse is mediated by the synaptonemal complex, which is composed of two lateral elements (formed mainly by the protein Synaptonemal complex protein 3-SYCP3, for example), linked to a central element (constituted by the protein Synaptonemal complex central element protein 3-SYCE3) (Zickler, 2006). These processes are controlled by an involved set of genes whose expression is regulated by epigenetic changes such as histone modifications and DNA methylation (Kota and Feil, 2010). Histone modifications associated with acetylation, such as acetylation at lysine 9 of histone H3 (H3K9ac) or acetylation at lysine 27 of histone H3 (H3K27ac), are related to regions of the genome with active gene expression (Wang et al., 2017). Differently, in heterochromatin, characterized by its high compactness, richness in repetitive DNA sequences, and few active genes, epigenetic markers linked to transcriptional repression are observed, such as di- and tri-methylated histone H3 lysine 9 (H3K9me2 and H3K9me3) (Grewal and Moazed, 2003). Therefore, understanding these epigenetic mechanisms may help to elucidate the molecular basis of meiotic recombination and synapse.

Considering the gap in knowledge about the genomic organization of repetitive DNA classes in Loricariidae, as well as regarding the meiotic cycle in fish, the present study aimed to perform the physical mapping of histone and snDNA U2 sequences in the species *Scobinancistrus aureatus, Scobinancistrus pariolispos* and *Spatuloricaria* sp. and evaluate the formation of synaptonemal complex and active chromatin during meiosis of two species of *Hypancistrus*. Additionally, we describe a new karyotype for the genus *Spatuloricaria*.

## RESULTS

**Fig. 1.**
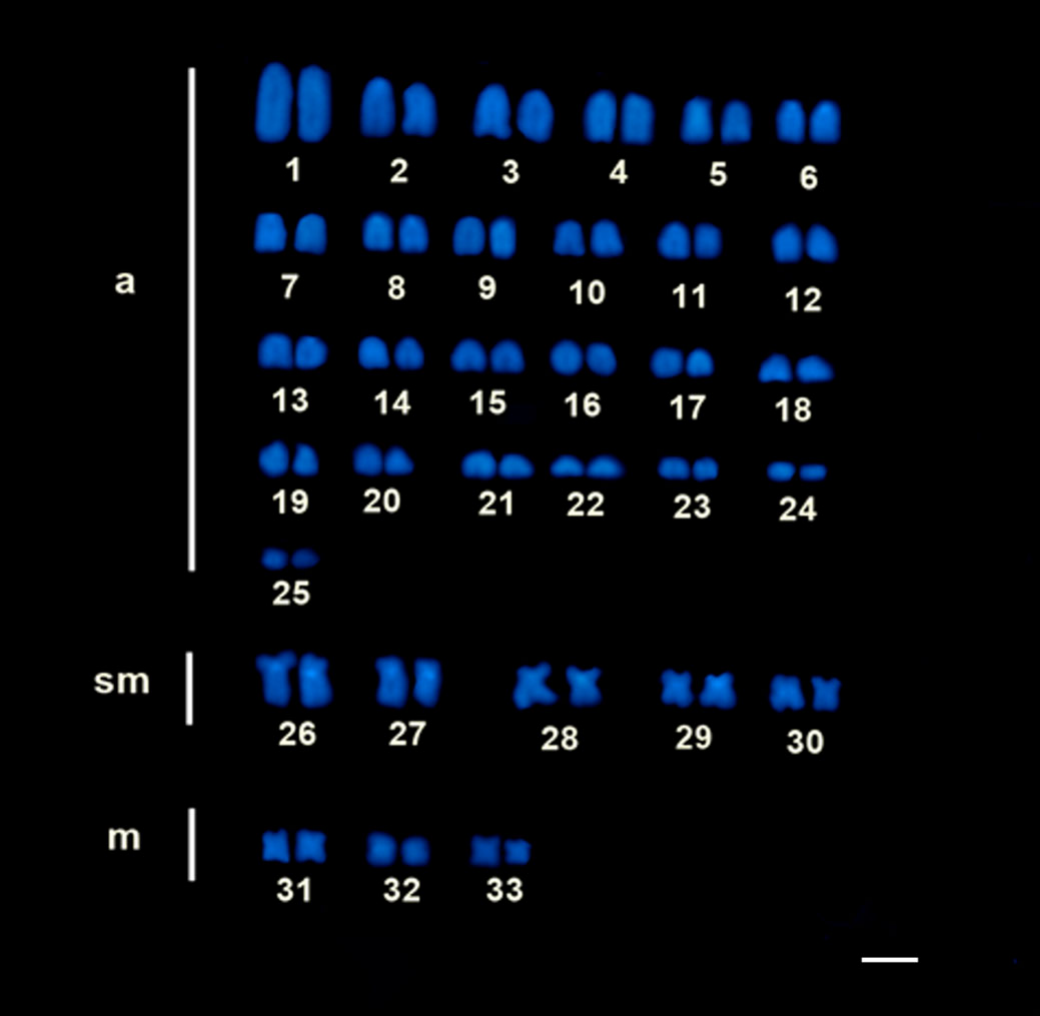
**Karyotype of *Spatuloricaria* sp (*n*=3).** DAPI-stained. Scale bar: 10 μm.

*Spatuloricaria* sp. presented diploid number 2*n*=66 and karyotypic formula composed of 50 acrocentric, 10 submetacentric and 6 metacentric chromosomes. The fundamental number was FN=82. Sex chromosomes with morphological differentiation were not observed for this species (Fig. 1). The karyotypes of both *Scobinancistrus* species (*S. pariolispos*: 2*n*=52, 22 metacentric, 20 submetacentric, 10 subtelocentric; *S. aureatus*: 2*n*=52, 24m-18sm-10st) and *Hypancistrus* (both *H. zebra* and *H. sp.* “pão” 2*n*=52, 40m/sm-12st) were previously described (Cardoso et al., 2013; da Silva et al., 2014; Ayres-Alves et al., 2017; Dos Santos et al., 2023). The fundamental number of *S. pariolispos* and *S. aureatus* was FN=94.

**Fig. 2.**
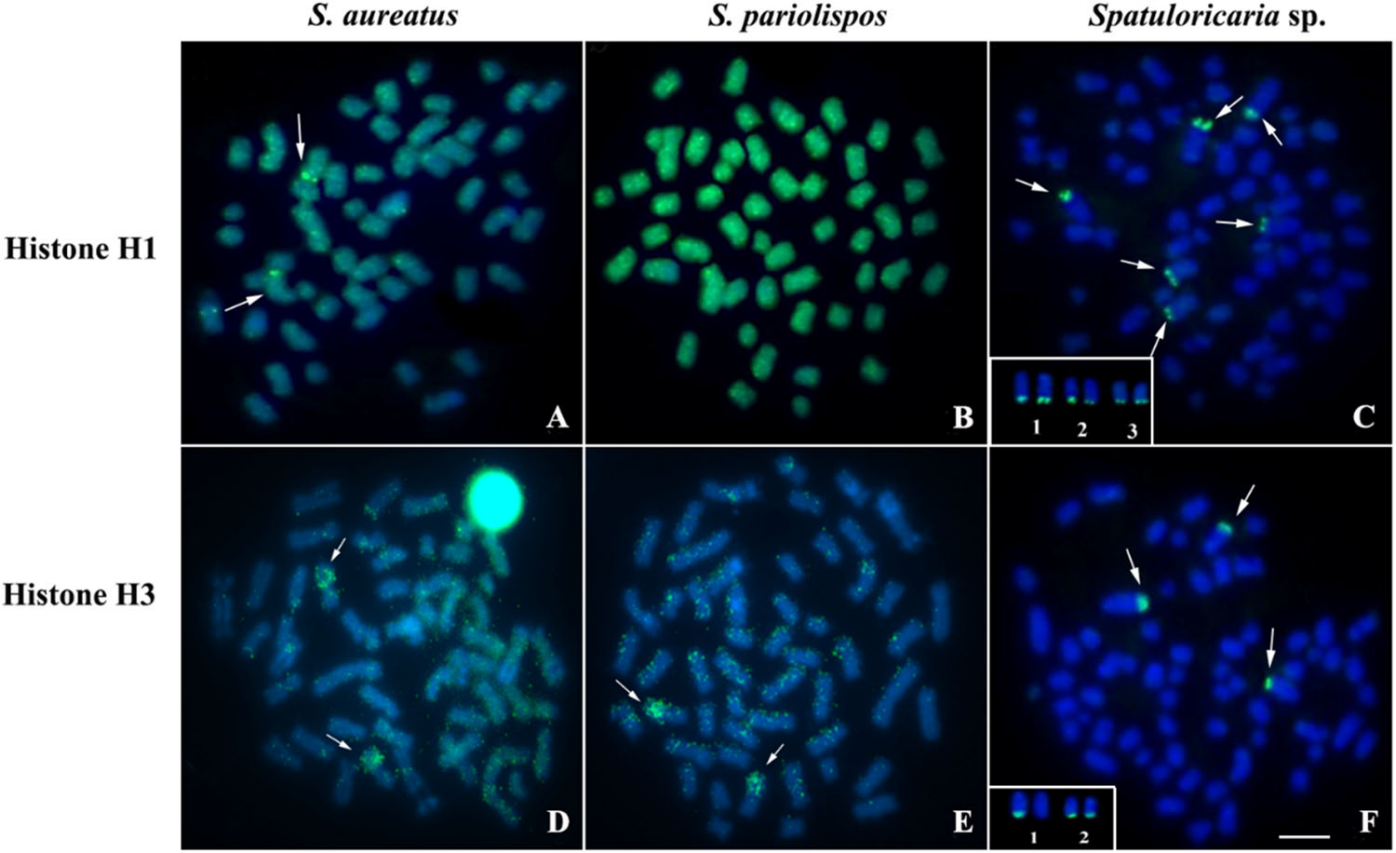
**Physical mapping of H1 histone genes.** (A) *S. aureatus* (B) *S. pariolispos* (C) *Spatuloricaria sp.*; H3 histone: (D) *S. aureatus* (E) *S. pariolispos* (F) *Spatuloricaria* sp. Scale bar: 10 μm.

FISH with histone H1 probe showed a dispersed pattern of this multigene along the chromosomes of *Scobinancistrus aureatus* (although clusters were observed on pair 3) and *Scobinancistrus pariolispos* (Fig. 2A and B). In *Spatuloricaria* sp. Histone H1 clusters were detected in the terminal region of chromosome pairs 1, 2 and 3 (Fig. 2C).

In *Scobinancistrus* the distribution pattern of histone H3 was similar to the markings found for histone H1. However, we recorded the presence of an interstitial cluster of histone H3 located in pair 4 of *S. aureatus* and *S. pariolispos* (Fig. 2D,E). In *Spatuloricaria* sp. blocks of histone H3 sequences were identified in the terminal regions of pairs 1 and 2. However, in pair 1 the marking was evident in only one of the chromosomes (Fig. 2F).

**Fig. 3.**
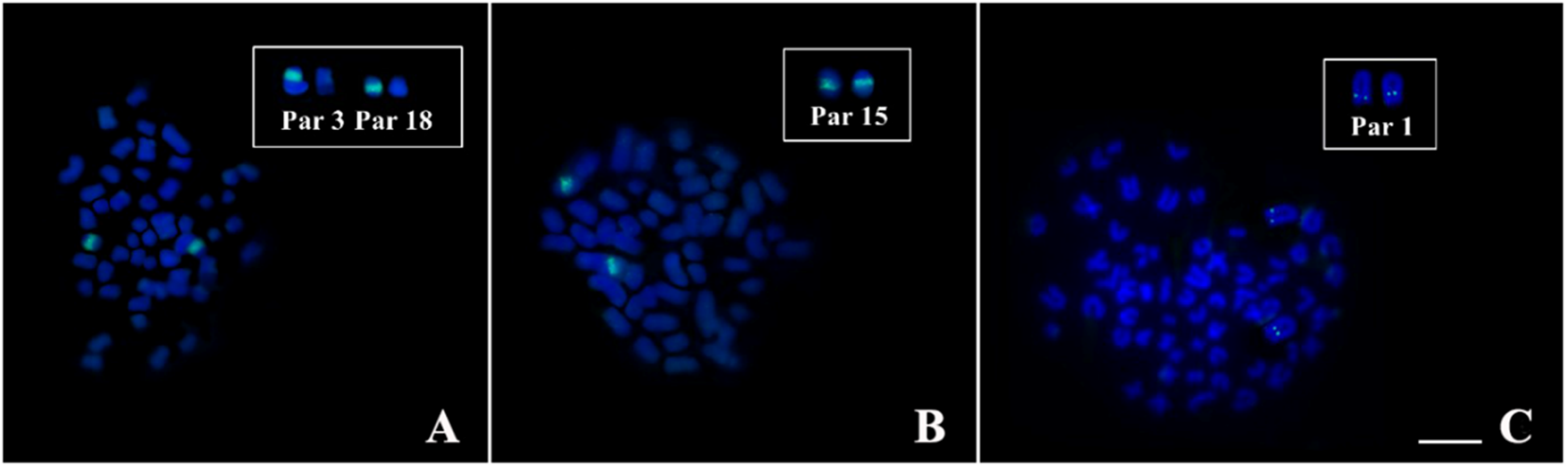
**Mapping of snDNA U2 in the genome.** (A) *S. aureatus* (B) *S. pariolispos* (C) *Spatuloricaria* sp. Scale bar=10 μm.

Hybridization signals of the U2 snDNA sequences were observed in chromosomes of different pairs in the species *S. aureatus*, which presented markings in the pericentromeric region in only one of the chromosomes of pairs 3 and 18 (Fig. 3A). In *S. pariolispos*, pericentromeric markings were observed in pair 15 (Fig. 3B). *Spatuloricaria* sp. showed marking signals in the interstitial region of the long arm of the pair 1 chromosomes (Fig. 3C).

**Fig. 4.**
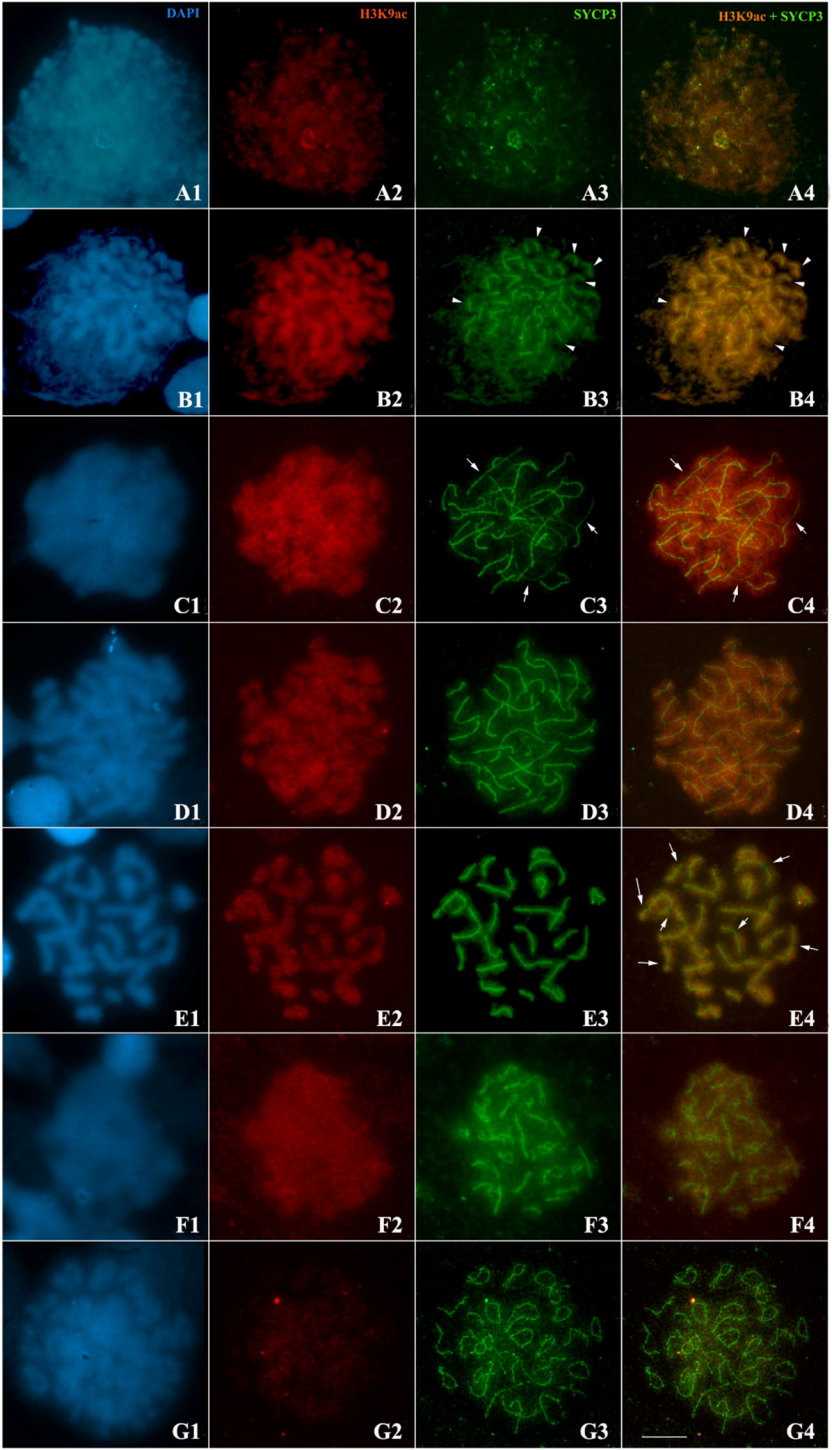
**Dynamics of H3K9ac-rich chromatin (red) and organization of the synaptonemal complex (SYCP3, green) in chromosomes (DAPI, blue) of prophase I of *H. zebra*.** (A1-A4) Leptotene; (B1-B4) early zygotene, arrowheads indicate synapsed chromosomal regions; (C1-C4) late zygotene, note the presence of H3K9ac uniformly in synapsed and non-synapsed regions (arrows); (D1-D4) early pachytene; (E1-E4) Late pachytene, arrows indicate H3K9ac negative regions; (F1-F4) early diplotene; (G1-G4) late diplotene. Scale bar: 10 μm.

Regarding prophase I in *Hypancistrus zebra*, at the end of leptotene, it was possible to observe the formation of short SYCP3 fragments (Fig. 4A3). In early zygotene, the synaptic process begins, and the synaptonemal complex axes are seen close to one of the poles of the nucleus (Fig. 4B3). In late zygotene, long asynaptic regions of the bivalents were visualized (Fig. 4C3). In the initial pachytene, most of the bivalents already have full synapses, however, some pairs still have asynaptic regions (Fig. 4D3). In late pachytene, all bivalents are fully synapsed (Fig. 4E3). In the pachytene/diplotene transition, lateral elements connected only at some points along its length were recorded, indicating disorganization of the synaptonemal complex (Fig. 4F3). In diakinesis, bivalents are joined only by crossing points (Fig. 4G3).

Immunolocalization experiments showed that during meiosis of *H. zebra,* H3K9ac is observed in leptotene in a diffuse form (Fig. 4A1-A4); however, at the beginning of zygotene there is a considerable increase in the intensity of this epigenetic mark, especially concentrated on the chromatin, in which axes of the synaptonemal complex initiate the synaptic process (Fig. 4B1-B4); in late zygotene (Fig. 4C1-C2) and early/late pachytene (Fig. 4D1-E4), H3K9ac is observed uniformly throughout the bivalents, except in some pericentromeric regions, identified by DAPI-negative staining (Fig. 4E4). During the pachytene/diplotene transition there is a decrease in the concentration of H3K9ac (Fig. 4F1-F4). In late diplotene, this epigenetic mark was not observed (Fig. 4G1-G4).

**Fig. 5.**
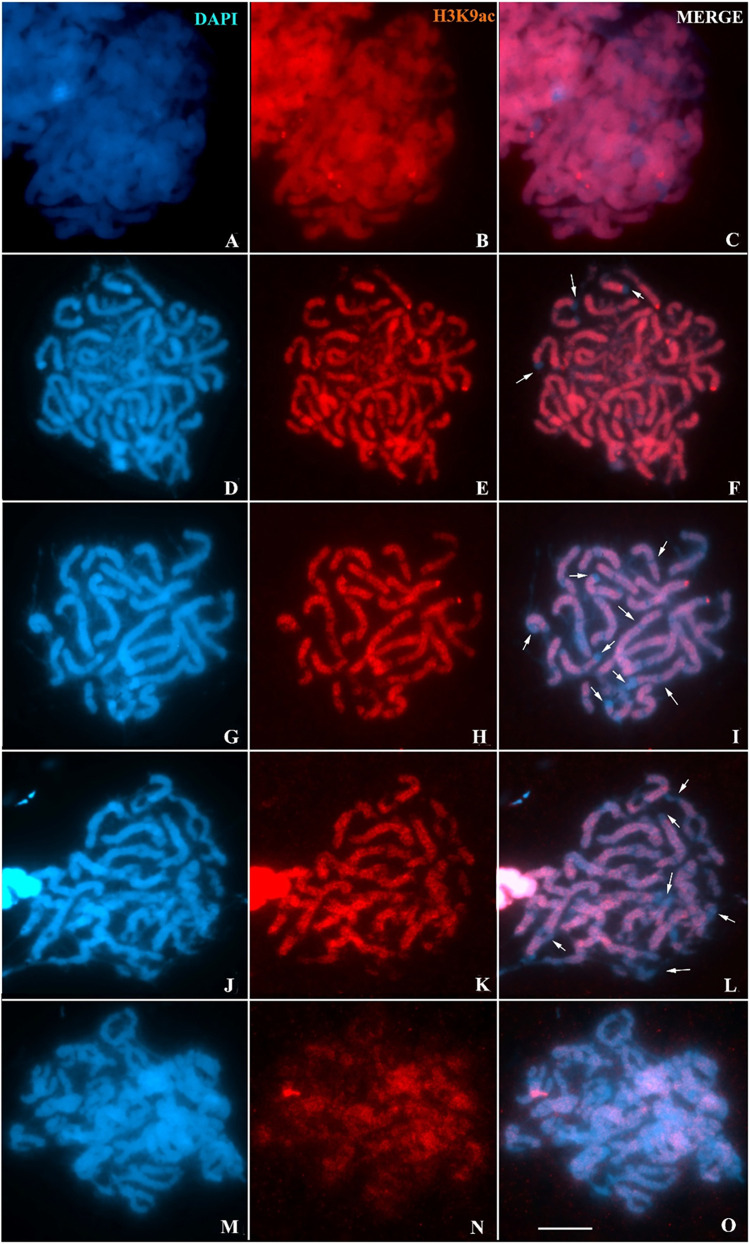
**Dynamics of H3K9ac-rich chromatin (red) in prophase I chromosomes (DAPI, blue) of *H.* sp. “pão”.** (A-C) late zygotene; (D-F) early pachytene; (G-I) late pachytene; (J-L) pachytene/diplotene transition; (M-O) diplotene. Arrows indicate DAPI+ heterochromatic regions. Scale bar: 10 µm.

Regarding the dynamics of H3K9ac during prophase I in *Hypancistrus* sp. “pão”, it is observed that at the end of zygotene (Fig. 5A-C) and at the beginning of pachytene (Fig. 5D-F) this epigenetic mark is found over most of the synapsed or asynaptic chromatin, except, in DAPI+ heterochromatic regions, and some pericentromeric regions identified by DAPI-negative staining. In late pachytene, the absence of H3K9ac in terminal or interstitial heterochromatin is more easily visualized due to increased chromosomal condensation (Fig. 5G-I). From the pachytene/diplotene transition (Fig. 5J-L), this pattern observed in previous phases is maintained, even with the beginning of disorganization of the synaptonemal complex. Finally, in diplotene there is a gradual decrease of H3K9ac in prophase chromosomes (Fig. 5M-O).

## DISCUSSION

### New karyotype in *Spatuloricaria* sp. from Amazonia

Among the members of the genus *Spatuloricaria* recognized in the literature to date, only one species (not determined) from the Xingu River was cytogenetically characterized (Ferreira et al., 2014). *Spatuloricaria* sp. (present study, Caripetuba River) presented a diploid number 2*n*=66, as well as the cytotype described in the works by Ferreira et al. (2014). However, despite the maintenance of 2*n*, both karyotypes show differences in the morphology of the chromosome pairs, reflecting in the fundamental number (NF). *Spatuloricaria* sp. (present study) presented NF=82 differently from NF=92 described by Ferreira et al. (2014). Both karyotypes are formed predominantly by acrocentric chromosomes. Thus, we suggest that this divergence is related to pericentric inversion rearrangements, which can change the chromosomal morphology without modifying the diploid number. Several studies demonstrate the important role of inversions during karyotype evolution of Loricariidae, as recorded in the genera *Ancistrus* (Mariotto et al., 2009), *Hypancistrus* (da Silva et al., 2014), *Loricariichthys* (Takagui et al., 2014) and *Rineloricaria* (Venturelli et al., 2021). Alternatively, we do not rule out the possibility that these differences are the result of centromeric repositioning, as demonstrated in other organisms (Han et al., 2009; Rocchi et al., 2012). Both mechanisms (inversions or centromeric repositioning) have the potential to form reproductive barriers between populations, contributing to speciation events (Lu and He, 2019).

### Chromosomal mapping of repetitive DNA in Loricariidae Amazon

Scattered marking signals evidenced by FISH for the Histone H1 gene in *S. aureatus* and *S. pariolispos* revealed a distribution pattern considered atypical for these markers, since most studies show these sequences organized in conserved clusters in relation to location, between related organisms, as observed in grasshopper species (Cabrero et al., 2009) and in other fish species (Pendás et al., 1994; Hashimoto et al., 2011). A similar result was found by Utsunomia et al. (2014) who mapped the H1-H4 histone genes in *Synbranchus marmoratus* and proposed that these sites are organized in small repeats abundant in the genome. Pucci et al. (2018) found transposable elements (ERV1 and Gypsy) inserted into H1-H4 histone sites in *Characidium* species, and proposed that these may have acted in the multiplication of copies of histone genes. In other vertebrates, molecular co-option of some mobile elements inserted into histone genes was reported (Cavalcante et al., 2020). Thus, it is plausible to suggest that, as proposed by Pucci et al. (2018), the dispersed pattern revealed in the present study resulted from histone H1 associations and transposable elements (TEs). Alternatively, the occurrence of other scattering factors of repetitive DNAs, such as circular DNAs, may have contributed to the mobilization of histone H1 genes in the *Scobinancistrus* genome (Cohen et al., 2003).

Our findings showed that in *Spatuloricaria* sp. histone H1-H3 genes may be colocalized, since FISH analysis showed the presence of conspicuous blocks of these multigenes in the terminal regions of pairs 1 (in only one homolog), 2 and 3 of the karyotype. This result agrees with the in tandem arrangement of histone genes, which tend to remain together on the same chromosomes (Cabrero et al., 2009). The colocalization of H1 and H3 histone genes in *Spatuloricaria* sp. may have a functional significance, as the intrachromosomal spatial proximity of neighbouring genes can promote co-expression due to the sharing of promoters, transcription factors and histone modifiers (Dai et al., 2014). Additionally, in *Spatuloricaria* sp. the absence of histone H1 sequences in one homologues of pair 1 can be explained by the occurrence of non-reciprocal translocation-type rearrangements or deletions (Pety et al., 2018).

Data on the physical location of snDNA U2 in Loricariidae is considered limited; in fish, two common patterns of genomic organization were recognized for this multigene: (I) forming in tandem arrays in one or more chromosome pairs or (II) randomly distributed throughout the genome (Úbeda-Manzanaro et al., 2010; Silva et al., 2015; Utsunomia et al., 2014). In the present study, a certain degree of conservation of the number of sites of this repetitive DNA was reported in the three analysed species; nevertheless, the occurrence of defective copies and pseudogenes not detected by FISH may exist in the genome of the three Siluriformes investigated, as recorded for *Aparaeiodon* sp. (Parondontidae) (Azambuja et al., 2022). In *S. aureatus*, U2 snDNA clusters showed localization on non-homologous chromosomes of pairs 3 and 18, similar to the findings by Santos da Silva et al. (2021) for U1 snDNA in *Ancistrus*. The pattern observed in *S. aureatus* agrees with the action of mechanisms that promote variability and dispersion of this sequence to different loci, such as ectopic recombination and chromosomal rearrangements (Dutrillaux and Dutrillaux, 2019; Louzada et al., 2020). The location of U2 snDNA in a heterochromatic block and its terminal position in *Scobinancistrus* may be important factors for the occurrence of these processes, similar to that described for 45S rDNA (Cazaux et al., 2011).

### Synapse and gene transcription during meiosis in *Hypancistrus*

The organization of the synaptonemal complex in *H. zebra* and *Hypancistrus* sp. “pão” is similar to patterns described in other species of teleost fish (Iwai et al., 2006; Blokhina et al., 2019; Cardoso et al., 2022); in most cases, synapse is complete during pachytene, even if late as observed between heterochromatic regions on the B chromosome of *Oreochromis niloticus* (Ocalewicz et al., 2009); this fact differs from meiosis in more basal vertebrates, such as Ciclostomata, in which the synapse is incomplete, especially close to telomeres (Matveevsky et al., 2023).

In mammals, there is a strong relationship between transcriptional activity during meiosis and synapse realization. In mice, transcription is inhibited in leptotene/zygotene, being subsequently reactivated during pachytene, and suppressed again from diplotene to the second meiotic division (Page et al., 2012). In this case, for transcriptional reactivation it is necessary that all bivalents complete the synaptic process. Recently, a study with *Lampetra fluviatilis* (Cyclostomata) using specific antibodies for RNA polymerase II, showed that the reactivation of transcription occurs along the zygotene, independently of the realization of the synapse (Matveevsky et al., 2023). In the present study, we used an anti-H3K9ac antibody as a marker of transcriptional activity, because this epigenetic modification is mainly related to the formation of active chromatin (Page et al., 2012; Yamada et al., 2013). Considering this fact, it can be concluded that our results agree with the expected pattern for the chromosomal distribution of this marker, since it was widely observed along the euchromatin in both species of *Hypancistrus*. In *Characidium gomesi*, H3K4m (another epigenetic marker related to active chromatin), showed a similar pattern in pachytene (Serrano et al., 2016). Our results suggest that transcriptional activity in *Hypancistrus* meiosis follows the pattern observed in *Lampetra fluviatilis*, since the first signs of H3K9ac are observed from the early zygotene, when extensive asynaptic regions are observed; this feature may be conserved among lower vertebrates.

The absence of H3K9ac in DAPI+ heterochromatic regions and in pericentromeric regions DAPI negative in the studied individuals shows low transcriptional activity in the constitutive heterochromatin of *Hypancistrus* during prophase I. This phenomenon was more easily visualized in *Hypancistrus* sp. “pão”, since this species has more conspicuous and relatively numerous heterochromatic blocks than *H. zebra* (da Silva et al., 2014; Cardoso et al., 2016). Similar results were observed in other animal taxa (Almeida et al., 2019). The low transcriptional activity in heterochromatin is necessary especially for the silencing of transposable elements, which can promote genomic instability (Cavaliere et al., 2020). Additionally, the absence of recombination in heterochromatic regions is a factor common to several eukaryotes, as crossing over between repetitive sequences can generate chromosomal rearrangements that are harmful to the genome (Nambiar and Smith, 2016). In *Oreochromis niloticus*, for example, a linkage map showed a total absence of recombination in the large heterochromatic block of pair 1 (Ocalewicz et al., 2009). This fact may partially explain the absence of H3K9ac in *Hypancistrus* heterochromatin, since this epigenetic mark is usually present in chromosomal loci that constitute recombination hotpots (Yamada et al., 2013).

To conclude, the chromosomal mapping performed in the present study showed that the Histone H1-H3 and U2 snDNA multigenic families present distinct patterns of organization in the karyotype of *Scobinancistrus* and *Spatuloricaria*, despite the conservation of the diploid number in these genera, suggesting different evolutionary histories of these sequences in the referred genomes. In addition, the results obtained allow us to distinguish the two species of *Scobinancistrus* at the chromosomal level, which, combined with data on the distribution of ribosomal genes and constitutive heterochromatin described previously, constitute excellent cytotaxonomic tools. The organization of the synaptonemal complex in *Hypancistrus* is similar to that observed in mammals, with pachytenes that present complete synapsis of all bivalents. However, transcriptional activity starts during zygotene and ceases only during diplotene, which indicates that activation of gene expression during prophase I is independent of the completion of full synapse in *Hypancistrus*. Furthermore, we show the occurrence of cytogenetic diversity in *Spatuloricaria*, with the description of a new cytotype, contributing to the understanding of the chromosomal evolution of this group.

## MATERIALS AND METHODS

### Samples

**Fig. 6.**
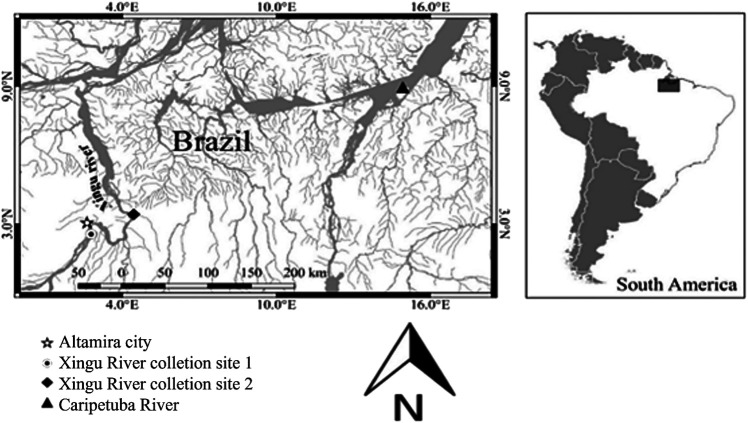
Sample collection locations for this study in municipalities Altamira and Abaetetuba, Pará, Brazil.

In the present study, male and female individuals of five species of the Loricariidae family were analysed: *Spatuloricaria* sp. (2 males and 1 female) from the Caripetuba River, Abaetetuba, Pará, Brazil (S=1°37′23.49″, W=48°55′33″) (Fig. 6); the others were collected in two sites along the Xingu River, municipality of Altamira, Pará, Brazil (Fig. 6): the first site located near the Belo Monte hydroelectric plant (S=03°06′12.8″, W=51°43′53.9″) where *Scobinancistrus aureatus* (2 males and 1 females), *Scobinancistrus pariolispos* (1 male and 3 females), *Hypancistrus zebra* (5 males) and *Hypancistrus* sp. “pão” (4 males) were collected; the second sampling site was close to the locality of “Gorgulho da Rita” (S=03°20′06.2″, W=52°10′32.9″), where 1 female of *S. aureatus* was collected. The sample size was appropriate considering the previous description of the karyotypes of these specimens (except *Spatuloricaria* sp.) and by comparison with similar studies in the current literature. The collections were authorized by the Chico Mendes Institute for Biodiversity Conservation (ICMBIO), license number 020/2005. The study was conducted in accordance with the Declaration of Helsinki, and approved by the Animal Use Ethics Committee of the Institute of Biological Sciences, UFPA (N°68-2015).

### FISH with probe of repetitive sequences in *Scobinancistrus* and *Spatuloricaria*

Metaphase chromosomes extracted from mitotic cells of the cephalic kidney according to Bertollo et al. (1972). The organization of karyotypes followed the classification system described by Levan et al. (1964) with chromosomes arranged in pairs and in decreasing order of size.

Genomic DNA was extracted from muscle tissue cells of the species *Scobinancistrus pariolispos* according to Sambrook et al. (1989). Polymerase Chain Reaction (PCR) using a set of primers listed in Table 2 performed amplification of the histones H1, H3 and U2 snDNA repetitive sequences. Each reaction was composed of: 100 ng/μl of genomic DNA; 2.5 μl of 10x buffer; 1.25 μl MgCl; 2.5 μl of DNTP mix (2 mM); 1 μl of each primer (10 mM); 0.3 μl of Taq polymerase (Invitrogen) and 16.25 μl of pure water. The thermal settings for each cycle were: 1 cycle of 95°C for 5 min; 35 cycles of 95°C for 1 min, 52°C–60°C for 50 s and 72°C for 2 min; 1 cycle of 72°C for 10 min; hold at 4°C. Probes were Nick-Translation labelled with DIG-Nick Translation Mix (Roche) for 11-dUTP-digoxigenin labelling.

**Table 2.**
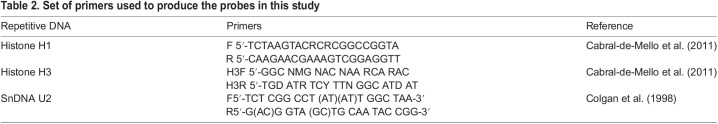
Set of primers used to produce the probes in this study

The FISH technique was performed according to the protocol described by Pinkel et al. (1986). Slides were treated with 1% Pepsin solution for 10 min and dehydrated in an alcohol battery (70%, 90% and 100%). Chromosomal DNA was denatured in 70% formamide at 65°C. The probes were denatured at 70°C. Hybridization took place overnight at 37°C. Probes were detected with anti-digoxigenin-FITC. Chromosomes were counterstained with DAPI containing Antifade VECTASHIELD (Vector).

### Meiotic analysis in *Hypancistrus zebra* and *H*. sp. “pão”

This analysis was carried only in *Hypancistrus* genus, as it was the only one to present male individuals with gonads in a suitable maturation stage for the study of meiosis. Male gonads kept in Hanks buffered saline solution for 10 min, and hypotonized in KCl 0.075 M for 20 min at room temperature. The gonads were subsequently macerated in 100 µM sucrose, to generate cellular suspension. About 60 µl of each solution was spread onto slides previously coated with 2% paraformaldehyde. The slides were kept in a humidified chamber for 2 h, washed in 0.4% Kodak Photo-flo solution, and stored at −80°C.

Immunodetection of meiotic proteins was performed according to the previous protocol Noronha et al. (2020). The primary antibodies used for immunolocalization of proteins included rabbit antibody anti-SYCP3 (ab15093, Abcam Ltd., UK, diluted 1:100) and histone H3 acetylated to lysine 9 -H3K9ac (Cell Signal, 9733S, diluted 1:50). Slides were washed in PBS, and blocked in 5% BSA, 0.1% Tween 20, for 30 min at room temperature. The slides were washed and incubated with primary antibodies for 1 h at 37°C in a humid chamber. After washing, the slides were incubated for 2 h at 37°C with rabbit anti-IgG conjugated to TRITC or FITC and diluted 1:100 in PBST. The slides were again washed in 1X PBST and counterstained with DAPI containing antifading Vectashield.
